# An optimised whole blood interleukin‐2 release assay is more sensitive than interferon‐γ ELISpot for detecting and quantifying gluten‐specific CD4^+^ T‐cell responses in coeliac disease

**DOI:** 10.1002/cti2.70063

**Published:** 2025-12-10

**Authors:** Olivia G Moscatelli, Amy K Russell, Lee M Henneken, Melinda Y Hardy, Jason A Tye‐Din

**Affiliations:** ^1^ Immunology Division The Walter and Eliza Hall Institute Parkville VIC Australia; ^2^ Department of Medical Biology University of Melbourne Parkville VIC Australia; ^3^ Department of Gastroenterology The Royal Melbourne Hospital Parkville VIC Australia

**Keywords:** coeliac disease, ELISpot, interleukin‐2, T cells

## Abstract

**Objectives:**

Detection of antigen‐specific T cells has been crucial for the study of autoimmune illnesses such as coeliac disease (CD). In CD, an oral gluten challenge is required to expand the rare population of circulating gluten‐specific T cells to detectable levels for IFN‐γ ELISpot detection. We evaluated interleukin (IL)‐2 detection as a simpler approach to identify gluten‐specific T cells in CD.

**Methods:**

HLA‐DQ2.5+‐treated CD adults (*n* = 10) were assessed before and 6–8 days after commencing a single‐bolus gluten challenge. Serum IL‐2 (GCIL‐2), whole blood IL‐2 (WBA) and peripheral blood mononuclear cell IFN‐γ ELISpot responses were evaluated. Functional effects of peptides encompassing epitopes of varying immunogenicity and pan‐HLA‐DQ blocking were tested.

**Results:**

GCIL‐2 was 90% sensitive in detecting CD. Day 6 post challenge was the optimal day for assessment. *In vitro* IL‐2 release showed higher sensitivity than IFN‐γ or IL‐10 release in the WBA. IL‐2 WBA had 80% sensitivity prior to gluten challenge, and 90% sensitivity at Day 6 post gluten challenge. IFN‐γ ELISpot was less sensitive: 20% at baseline and 40% at Day 6 after gluten challenge. Pan‐HLA‐DQ blocking reduced IL‐2 WBA responses by 89–91%, and the assay accurately ranked gluten peptide immunogenicity consistent with published data.

**Conclusions:**

The IL‐2 WBA provides superior sensitivity over IFN‐γ ELISpot for detecting gluten‐specific T cells. It can measure the functional blockade of HLA and accurately reflects gluten peptide immunogenicity hierarchies. The IL‐2 WBA supports immunomonitoring and T‐cell epitope mapping in CD and offers broad research and clinical application beyond CD.

## Graphical abstract

The ELISpot assay is widely used for measuring antigen‐specific lymphocyte activation. Here, we demonstrate a novel whole blood interleukin‐2 release assay that shows superior sensitivity to IFN‐γ ELISpot and can accurately measure T‐cell epitope immunogenicity and HLA‐dependent responses. The assay offers broad clinical and research utility beyond coeliac disease.

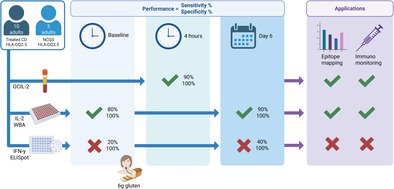

## Introduction

The capacity to measure and characterise antigen‐specific lymphocyte responses has revolutionised our understanding of disease mechanisms in cancer, infections and autoimmune conditions, while also advancing vaccine development. Current methodologies encompass flow cytometry‐based approaches (including peptide–MHC tetramers, intracellular cytokine staining and proliferation assays), functional assessments (such as ELISpot and antigen‐stimulated cytokine release assays) and more recently technologies such as single‐cell RNA sequencing.^1^, ^2^, ^3^, ^4^, ^5^, ^6^ Each method offers distinct advantages for measuring T‐ or B‐cell responses, from direct visualisation of antigen‐specific cells to quantification of their functional outputs, although all have inherent technical and practical limitations. The critical importance of these immunological tools has been highlighted by the COVID‐19 pandemic and the rapid development of immunotherapeutics across multiple diseases.^5^, ^7^

The ELISpot assay offers exceptional sensitivity, detecting as few as 1–5 cytokine‐producing cells per 100 000 cells depending on the application. This sensitivity level parallels that of RT‐PCR analysis, but with the advantage of detecting functionally relevant secreted proteins rather than mRNA transcripts. Its minimal sample requirements, single‐cell resolution and high reproducibility make it particularly informative for vaccine development and immunomonitoring studies. Despite limitations in multiplexing capability, the ELISpot assay remains a highly favored method because of its high sensitivity in detecting low‐frequency antigen‐specific T‐ and B‐cell responses, with validated assays used for both clinical trial endpoints and diagnostic testing, for example of tuberculosis.^8^

In coeliac disease (CD), a prevalent CD4^+^ T‐cell‐driven autoimmune‐like enteropathy triggered by dietary gluten from wheat, rye and barley, the ELISpot assay has provided fundamental insights through comprehensive T‐cell epitope mapping of gluten.^9^, ^10^ It has also advanced therapeutic development by supporting pre‐clinical CD drug discovery, including establishing therapeutic proof‐of‐concept^11^, ^12^ and evaluating the efficacy of novel CD drugs,^13^, ^14^ as well as enabling the design of ultra‐low gluten cereals.^15^ However, despite its high sensitivity, a major limitation of the ELISpot approach is that CD patients need to consume oral gluten for 3 days to expand the circulating gluten‐specific T‐cell pool to a detectable level.^16^, ^17^, ^18^

More recently, we and others have shown that serum interleukin (IL)‐2 after single‐dose oral gluten challenge is a highly sensitive and specific marker of pathogenic gluten‐specific T cells and the presence of CD.^19^, ^20^, ^21^, ^22^, ^23^, ^24^, ^25^ The elevation of IL‐2 in blood correlates with the severity of gluten‐induced symptoms and is produced by the activated gluten‐specific CD4^+^ T cell.^12^, ^20^ A whole blood assay (WBA) that relies on *in vitro* release of IL‐2 following incubation of blood with gluten peptides has been subsequently developed.^26^, ^27^ This approach overcomes the need for patients to undergo an oral gluten challenge and demonstrates high sensitivity and specificity for CD with promising potential as a blood‐based diagnostic for CD.^24^

Here, we compared the sensitivity and performance of the IL‐2 WBA against the IFN‐γ ELISpot assay after no or minimal (single‐dose) gluten ingestion. Our approach was intentionally designed to evaluate assay performance under less burdensome conditions for participants, though this abbreviated gluten challenge is shorter than the standard 3‐day protocol typically employed to maximise IFN‐γ ELISpot assay sensitivity in CD. We also assessed whether the IL‐2 WBA can support T‐cell epitope mapping and monitoring the functional status of the gluten‐specific T cell.

## Results

### Participant demographics

Ten adults with CD following a strict gluten‐free diet (treated CD) and three adults with self‐reported non‐coeliac gluten sensitivity (NCGS) were recruited (Table 1). Nine of 10 treated CD and 2/3 NCGS participants were female. All the CD participants were heterozygous for the CD‐susceptibility genotype HLA‐DQ2.5, whereas two of the NCGS participants carried HLA‐DQ2.5 and the remaining person carried HLA‐DQ7.

**Table 1:** Patient demographics

	Treated CD (*n* = 10)	NCGS (*n* = 3)
Sex	9 female; 1 male	2 female; 1 male
Age, years, median (range)	53 (24–70)	67 (30–70)
GFD, years, median (range)	18 (4–53)	14 (5–33)
Positive TTG/DGP serology, *n* (%)	0 (0%)	0 (0%)
HLA‐DQ2.5, *n* (%)	10 (100%)	2 (67%)
Anticipated symptoms, *n* (%)	10 (100%)	3/3 (100%)
Experienced symptoms, *n* (%)	10 (100%)	2/3 (67%)
Symptom severity, median (range)	5/10 (2/10–8/10)	4/10 (0/10–7/10)

CD, coeliac disease; GFD, gluten‐free diet; HLA, human leukocyte antigen; NCGS, non‐coeliac gluten sensitive; TTG, tissue transglutaminase.

### Optimisation of peptide concentrations and sampling time points for IL‐2 WBA and IFN‐γ ELISpot

To establish the optimal protocol for the IL‐2 WBA and IFN‐γ ELISpot assays following a single‐dose gluten challenge, a range of peptide concentrations and post‐challenge sampling timepoints was tested. A 3‐day oral gluten challenge has been shown to expand the circulating pool of gluten‐specific CD4^+^ T cells by Days 6–8.^9^, ^16^, ^17^, ^28^, ^29^ We postulated less peptide would be required for the WBA than for the ELISpot given the higher sensitivity of the WBA and that Day 6 would be the optimal time point for blood collection post‐challenge, similar to after the 3‐day challenge.

**Figure 1:**
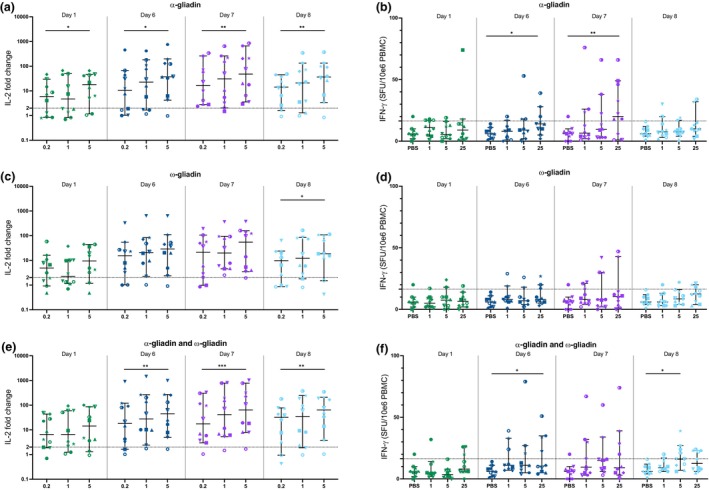
IL‐2 WBA and IFN‐γ ELISpot responses across concentrations and days in treated CD. IL‐2 fold change (peptide/PBS) from WBA stimulation with PBS or 0.2, 1 or 5 μg mL^−1^ of peptide (left) and IFN‐γ SFU (per million PBMC) from ELISpot stimulation with PBS or 1, 5 or 25 μg mL^−1^ of peptide (right) are shown for α‐gliadin (**a, b**), ω‐gliadin (**c, d**), and an equimolar pool of α‐gliadin and ω‐gliadin (**e, f**) in treated coeliac disease (CD) participants (*n* = 10) at baseline (Day 1) and Days 6–8 post gluten challenge. Median and 95% confidence intervals are shown. Dashed lines indicate calculated cutoffs. Friedman tests were performed to compare peptide concentrations; *P < 0.05, **P < 0.01, ***P < 0.001, ****P < 0.0001.

Three peptide concentrations were assessed: 0.2, 1 and 5 μg mL^−1^ for WBA, and 1, 5 and 25 μg mL^−1^ for ELISpot.^26^ Blood samples were collected at baseline (Day 1, pre‐challenge) and on Days 6, 7 and 8 post challenge. At the selected peptide concentrations, positive responses (> 2‐fold change in IL‐2; peptide compared to PBS) were observed in the majority of CD participants (50–100%) across all time points and for each gliadin peptide tested (Figure 1a, c and e and Supplementary figure 1).

Dose‐dependent IL‐2 secretion was observed in the WBA across all time points, with the most pronounced effect at baseline where positive responses increased from 50% to 60% at 0.2 μg mL^−1^ to 80% at 5 μg mL^−1^. In contrast, the IFN‐γ ELISpot did not detect positive responses above the cutoff threshold in the majority (10–70%) of matched samples, despite testing higher peptide concentrations (Figure 1b, d and f). For both assays, the highest tested concentrations yielded optimal sensitivity, demonstrating the maximum proportion of positive responses. Notably, the WBA showed robust responses (60–90% positive) even at the lowest peptide concentration tested (0.2 μg mL^−1^), which was 125‐fold lower than the highest concentration used in the ELISpot assay (25 μg mL^−1^). The ELISpot, however, failed to consistently generate robust responses even at this higher concentration.

Peak median IL‐2 responses were detected on Days 6 and 7 post gluten challenge (Supplementary figure 1), with these time points showing a higher proportion of positive responses (90–100%) compared to Day 8 (80–90%) at the optimal peptide concentration (5 μg mL^−1^). The median IFN‐γ ELISpot responses were mostly below the established cutoff threshold, preventing definitive determination of the optimal time point. One participant was classified as a non‐responder because of elevated PBS control responses above cutoff without significantly higher peptide‐specific responses. IL‐2 responses were absent in all NCGS participants (Supplementary figure 2), supporting the assay's disease specificity for CD.

These data demonstrate that a single oral gluten challenge enhances *in vitro* IL‐2 and IFN‐γ production that is specific to CD and is highest on Days 6 and 7 after starting the challenge. Based on these results, baseline and Day 6 time points were selected for subsequent analyses. Further, IFN‐γ ELISpot responses following a single‐dose gluten challenge are less consistent than after the usual 3‐day challenge protocols.^26^, ^30^ For direct comparison between assays, the highest tested concentrations were selected as optimal: 25 μg mL^−1^ for the IFN‐γ ELISpot and 5 μg mL^−1^ for the IL‐2 WBA.

### Enhanced sensitivity of IL‐2 detection in the whole blood assay

**Figure 2:**
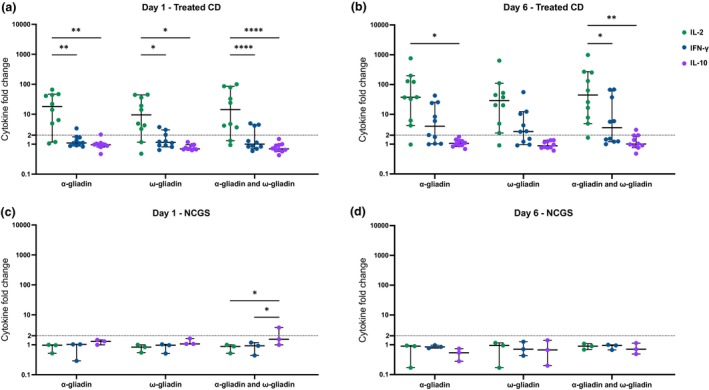
Comparison of IL‐2, IFN‐γ and IL‐10 WBA responses in treated CD and NCGS. IL‐2 (green), IFN‐γ (blue) and IL‐10 (purple) fold change from WBA stimulation with 5 μg mL^−1^ of α‐gliadin, ω‐gliadin, and α‐gliadin and ω‐gliadin at Day 1 (left) and Day 6 (right) in treated coeliac disease (CD) participants (top, *n* = 10, **a, b**) and non‐coeliac gluten sensitive (NCGS, **c, d**) participants (bottom, *n* = 3). Dashed lines indicate a twofold cutoff. Median and 95% confidence intervals are shown. Friedman tests were performed to compare cytokine responses; *P < 0.05, **P < 0.01, ***P < 0.001, ****P < 0.0001.

To identify the optimal cytokine in the WBA, other cytokines significantly elevated in serum following single‐dose oral gluten challenge, IFN‐γ and IL‐10, were assessed. At baseline (Day 1), peptides containing immunodominant α‐gliadin and ω‐gliadin T‐cell epitopes induced significantly higher fold changes in IL‐2 compared to IFN‐γ and IL‐10 (Figure 2). Positive responses above the cutoff were detected in the majority of participants on Day 1 (80%), only with IL‐2 measurement. By Day 6 post challenge, IL‐2 demonstrated the highest detection rate (90% positive responses), followed by IFN‐γ (50–60%), while IL‐10 responses remained predominantly below the cutoff. Both IL‐2 and IFN‐γ showed increased proportions of positive responses on Day 6 compared to baseline across all tested peptides. These findings establish IL‐2 release in the WBA as the most sensitive readout of gluten‐specific T‐cell activation.

### Comparative analysis of GCIL‐2 and IL‐2 WBA sensitivity in gluten‐induced cytokine assays

Following optimisation of peptide concentrations and post‐challenge sampling time points for both WBA and ELISpot, assay performance was compared across cohorts, with *in vitro* assays focussing on responses to the α + ω‐gliadin peptide pool (Table 2). The GCIL‐2 (based on *in vivo* serum IL‐2 release) showed 90% sensitivity for detecting treated CD. Among *in vitro* assays, the IL‐2 WBA achieved equivalent sensitivity (90%) on Day 6 post challenge, while maintaining 80% sensitivity at baseline. Notably, CD participant #8, initially negative for IL‐2 WBA at baseline, converted to positive by Day 6 post challenge, suggesting that gluten‐induced expansion of circulating gluten‐specific T cells may be necessary to reach the level of detection in some individuals. The IFN‐γ WBA and ELISpot demonstrated substantially lower sensitivities: at baseline, 30% and 20% respectively, increasing to 50% and 40% by Day 6 post challenge. CD participant #9, who tested negative across all assays, is undergoing diagnostic re‐evaluation.

**Table 2:** GCIL‐2, WBA and ELISpot responses for each participant

	GCIL‐2	D1 WBA IL‐2^a^	D6 WBA IL‐2	D1 WBA IFN‐γ	D6 WBA IFN‐γ	D1 IFN‐γ ELISpot	D6 IFN‐γ ELISpot
CD 1	Pos	Pos	Pos	**Neg**	**Neg**	**Neg**	**Neg**
CD 2	Pos	Pos	Pos	**Neg**	**Neg**	**Neg**	**Neg**
CD 3	Pos	Pos	Pos	**Neg**	**Neg**	**Neg**	**Neg**
CD 4	Pos	Pos	Pos	**Neg**	Pos	**Neg**	**Neg**
CD 5	Pos	Pos	Pos	Pos	Pos	**Neg**	**Neg**
CD 6	Pos	Pos	Pos	Pos	Pos	**Neg**	Pos
CD 7	Pos	Pos	Pos	Pos	Pos	Pos	Pos
CD 8	Pos	**Neg**	Pos	**Neg**	**Neg**	Pos	Pos
CD 9	**Neg**	**Neg**	**Neg**	**Neg**	**Neg**	**Neg**	**Neg**
CD 10	Pos	Pos	Pos	**Neg**	Pos	**Neg**	Pos
Non‐CD 1	Neg	Neg	Neg	Neg	Neg	Neg	Neg
Non‐CD 2	Neg	Neg	Neg	Neg	Neg	Neg	Neg
Non‐CD 3	Neg	Neg	Neg	Neg	Neg	ND	ND
Sensitivity	90%	80%	90%	30%	50%	20%	40%

CD, coeliac disease; ELISpot, enzyme linked immunospot; GCIL‐2, gluten challenge IL‐2; IFN‐γ, interferon‐γ; IL‐2, interleukin 2; ND, not done; WBA, whole blood assay.

Results disconcordant with diagnosis are indicated in bold red.

^a^ *In vitro* assay results refer to responses to the equimolar pool of α‐gliadin and ω‐gliadin at the optimised concentration for each assay.

### IL‐2 WBA accurately reflects epitope hierarchy and the effects of immune modulation

The ability of IL‐2 WBA to establish a ranking of T‐cell epitopes based on their immunogenicity (epitope hierarchy) was evaluated in comparison with the traditional approach using IFN‐γ ELISpot.^9^ The analysis focussed on peptides containing immunodominant wheat α‐gliadin and ω‐gliadin epitopes, subdominant wheat γ‐gliadin epitope, and immunodominant barley Hor3a epitope (Supplementary table 1). IL‐2 WBA was compared to IFN‐γ ELISpot following single‐dose gluten challenge.

**Figure 3:**
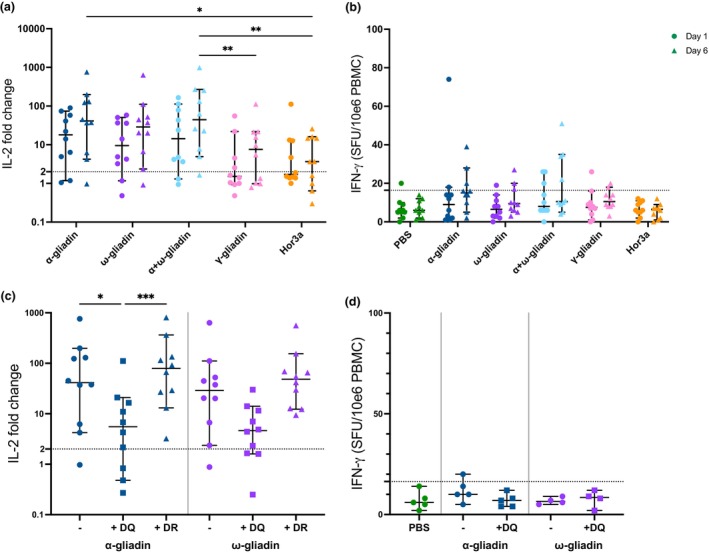
IL‐2 WBA reflects epitope hierarchy and immune modulation. IL‐2 fold change (peptide/PBS) from WBA stimulation with 5 μg mL^−1^ of peptide (left) and IFN‐γ SFU (per million PBMC) from ELISpot stimulation with 25 μg mL^−1^ of peptide (right) in treated coeliac disease (CD) participants (*n* = 10) at baseline (circle) and Day 6 (triangle) post gluten challenge (**a, b**). Responses to stimulation with PBS, α‐gliadin, ω‐gliadin, α‐gliadin and ω‐gliadin, γ‐gliadin and hor3a are shown. Responses to stimulation with α‐gliadin (blue) or ω‐gliadin (purple) alone (−, circle), with anti‐HLA‐DQ (+DQ, square), or with anti‐HLA‐DR (+DR, triangle) are shown (**c, d**, *n* = 4/5 for ELISpot). Dashed lines indicate calculated cut‐offs. Median and 95% confidence intervals are shown. Friedman tests were performed to compare response to different stimulation conditions; *P < 0.05, **P < 0.01, ***P < 0.001.

At baseline, IL‐2 responses to α‐gliadin (median: 18.04‐fold change, 2.34 pg mL^−1^), ω‐gliadin (median: 9.48‐fold change, 1.45 pg mL^−1^) and the α + ω‐gliadin pool (median: 14.31‐fold change, 2.07 pg mL^−1^) were more than 10‐fold higher than responses to γ‐gliadin (median: 1.52‐fold change, 0.014 pg mL^−1^) and Hor3a (median: 1.71‐fold change, 0.17 pg mL^−1^; Figure 3a and Supplementary figure 3A). At Day 6, the α + ω‐gliadin pool elicited significantly higher IL‐2 responses (median: 44.35‐fold change, 7.23 pg mL^−1^) compared to γ‐gliadin (median: 7.58‐fold change, 1.07 pg mL^−1^; *P* = 0.002 and 0.012) and Hor3a (median: 3.66‐fold change, 0.57 pg mL^−1^; *P* = 0.0008 and 0.004; Figure 3a and Supplementary figure 3A). The IL‐2 response to the α + ω‐gliadin pool was not significantly higher than to α‐gliadin or ω‐gliadin alone, demonstrating no additive effect. The IFN‐γ ELISpot showed no significant differences between peptides on either Day 1 or 6, though the α + ω‐gliadin pool elicited the highest number of positive responses (3 or 4/10 participants) compared to Hor3a (0/10; Figure 3b). The IL‐2 WBA detected responses to the subdominant γ‐gliadin epitope, which were not detectable by IFN‐γ ELISpot.

To evaluate the utility of IL‐2 WBA in assessing immunomodulatory effects, HLA‐DQ‐mediated antigen presentation was blocked using anti‐HLA‐DQ antibody (SPVL3), with anti‐HLA‐DR antibody (LB3‐1) serving as a control. Anti‐HLA‐DQ treatment significantly reduced IL‐2 WBA responses: α‐gliadin responses decreased by 91% (median fold change: 5.53 vs 41.34, *P* = 0.012; median concentration: 0.97 pg mL^−1^ vs 5.83 pg mL^−1^, *P* = 0.040) and ω‐gliadin responses by 89% (median fold change: 4.63 vs 28.91, *P* = ns; median concentration: 0.50 pg mL^−1^ vs 4.61 pg mL^−1^, *P* = ns; Figure 3c and Supplementary figure 3B). Anti‐HLA‐DR treatment showed no effect on IL‐2 responses to either peptide. IFN‐γ ELISpot analysis of HLA‐DQ blocking in a subset of treated CD participants (*n* = 4 or 5) showed no significant reduction, likely because of insufficient peptide responses (Figure 3d). These data confirm the IL‐2 WBA as a sensitive tool for detecting epitope hierarchies and the effects of T‐cell‐directed immunomodulation, outperforming the IFN‐γ ELISpot in single‐day challenge protocols.

### IL‐2 secretion but not symptoms distinguishes CD from NCGS

**Figure 4:**
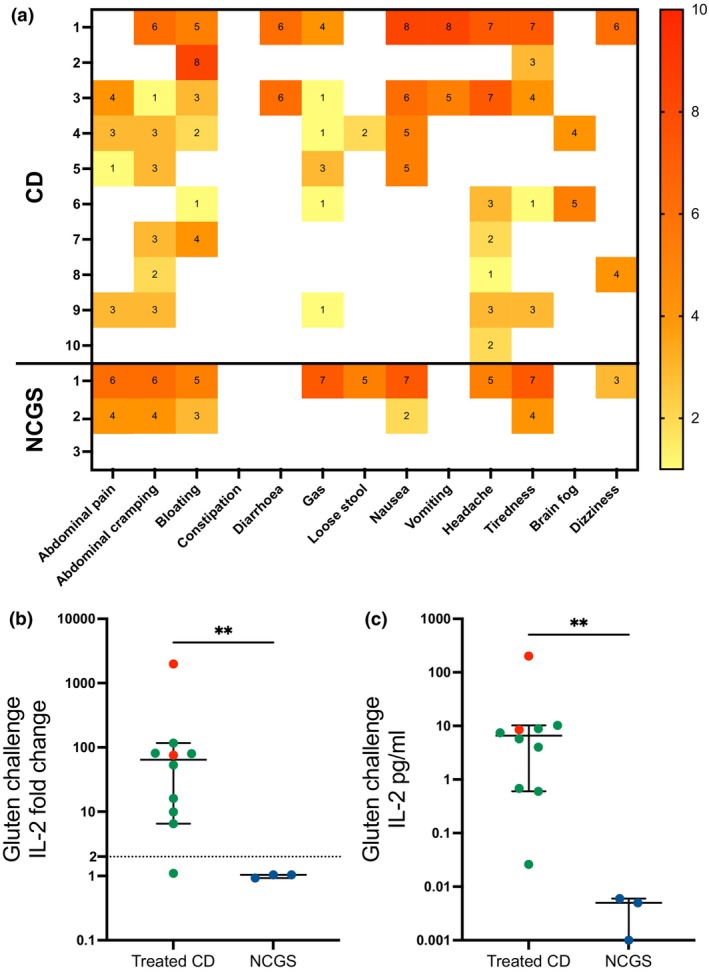
Oral gluten challenge serum IL‐2 response and symptoms. Heatmap of patient‐reported symptom scores after single bolus gluten challenge are shown for 10 treated coeliac disease (CD) and three non‐coeliac gluten sensitive (NCGS) participants (**a**). Maximum reported severity scores are shown, ranging from 0 (no symptoms; white) to 10 (severe symptoms; red). Serum IL‐2 fold change (**b**) and concentration (**c**, 4 h compared to 0 h) are shown. Participants that vomited following gluten challenge are indicated in red. A 2‐fold cut‐off is indicated by the dashed line. IL‐2 concentrations below zero were set at 0.001 to allow graphing on a log10 scale. Median and 95% confidence intervals are shown. Mann–Whitney *T* tests were performed; **P < 0.01.

Symptom anticipation and experience following gluten challenge were prevalent in both treated CD (10/10) and NCGS participants (2/3 experienced symptoms; 3/3 anticipated symptoms). Overall symptom severity was comparable between cohorts (median severity: CD 5/10, NCGS 4.5/10; Figure 4a). The CD cohort primarily reported abdominal cramping (7/10, median severity 3/10), headaches (7/10, median severity 3.6/10), bloating (6/10, median severity 3.8/10) and gas (6/10, median severity 1.8/10). Symptomatic NCGS participants experienced abdominal pain and cramping (median severity 5/10), bloating (median severity 4/10), nausea (median severity 4.5/10) and tiredness (median severity 5.5/10). In contrast to symptoms, gluten challenge‐induced serum IL‐2 (GCIL‐2) differentiated patient groups. Both GCIL‐2 fold change and concentration were significantly higher in CD participants (median: 64.4‐fold, 6.61 pg mL^−1^) compared to NCGS participants (median: 1.04‐fold, 0.005 pg mL^−1^; Figure 4b and c). GCIL‐2 had 90% sensitivity (9/10) for detecting the gluten‐specific immune response in treated CD.

## Discussion

The ELISpot assay has been instrumental in immunology research, offering exceptional sensitivity and reproducibility for detecting antigen‐specific immune responses across various diseases. Its capacity to assess gluten‐specific T cells in CD has provided fundamental insights into CD pathogenesis^16^, ^28^, ^31^, ^32^, ^33^ and supported immune discovery such as comprehensive T‐cell epitope mapping.^9^ Further, it has guided the design of ultra‐low gluten cereals^15^ and served as proof‐of‐concept and efficacy endpoints in both pre‐clinical drug discovery and CD therapeutic development.^11^, ^12^, ^13^, ^14^ Historically, detection of the rare, circulating CD4^+^ gluten‐specific T cells in CD was limited to MHC‐tetramers^34^ and IFN‐γ ELISpot following a 3‐day gluten challenge.^16^ Use of HLA‐DQ2.5‐gluten‐peptide tetramers is a highly sensitive approach to detect the effector memory CD4^+^ T cells specific for gluten and indicates their frequency is approximately 1 per 100 000 CD4^+^ T cells in CD and negligible in individuals without CD.^29^, ^34^ Unfortunately, peptide–MHC tetramers are technically demanding, require proprietary reagents, and demand large blood volumes.^28^, ^34^ IFN‐γ ELISpot is able to indirectly detect these rare cells but requires a gluten challenge to expand the circulating pool of gluten‐specific T cells.^29^ While shortening or eliminating the gluten challenge period would enhance patient recruitment for research and clinical trials, our findings clearly demonstrate that reducing the challenge from 3 days to a single dose substantially compromises the assay's ability to detect gluten‐specific T cells.

In this study, we confirm that a novel *in vitro* approach to detect gluten‐specific T cells through an indirect method, based on whole blood IL‐2 release using an ultrasensitive chemiluminescent platform, has high sensitivity and performs favorably compared to the traditional IFN‐γ ELISpot. We confirmed that a single‐dose oral gluten challenge induces a strong and rapid release of IL‐2 in the serum, with high sensitivity (90%) for CD, in line with ours and others' findings.^19^, ^20^, ^21^, ^22^, ^23^, ^24^, ^29^ Notably, we showed the *in vitro* IL‐2 WBA had 80% sensitivity prior to any gluten challenge and 90% sensitivity on Day 6 post single‐dose gluten challenge, comparable to the *in vivo* GCIL‐2 performance. These findings are striking when compared to IFN‐γ ELISpot, which has 20% sensitivity prior to gluten challenge and 40% after single‐dose gluten challenge. It is important to note that prior studies employing IFN‐γ ELISpot in CD have typically used a 3‐day gluten challenge, which shows a sensitivity for detecting gluten‐specific T cells after wheat gluten ingestion of 83–85%.^26^, ^30^ Consistent with previous studies, we showed that IL‐2 release was significantly higher than IFN‐γ release.^26^, ^27^ We postulate this may relate to lower background levels of IL‐2, resulting in a greater fold change upon stimulation, or differences in the kinetics of cytokine release. IL‐2 is produced rapidly and transiently upon antigen recognition, making it an early and sensitive marker of gluten‐specific CD4^+^ T‐cell activation.^20^ In contrast, IFN‐γ is commonly associated with the chronic mucosal lesion in CD and may require more prolonged or sustained T‐cell stimulation for its production.^35^ Thus, in contrast to the IL‐2 WBA, both the IFN‐γ ELISpot and IFN‐γ WBA lack sufficient sensitivity to detect gluten‐specific T cells after minimal or no gluten exposure.

The IL‐2 WBA before and after gluten challenge accurately reflected the published T‐cell epitope hierarchy,^9^ advancing prior data that supports its value as a T‐cell epitope mapping tool.^26^ This assay could potentially support simultaneous screening of T‐cell responses to wheat, barley and rye proteins without the current need for separate cereal challenges.^9^, ^31^, ^32^ Further, the assay provides a functional readout on the impact of immune modulators targeting the gluten‐specific T cell, making it valuable as a monitoring tool or efficacy readout for novel therapies aiming to reduce the pathogenic gluten‐specific T‐cell response. In the format we employed here, practical advantages include minimal blood requirements (0.25–1 mL per 1–4 replicate wells), pre‐prepared frozen antigen templates enabling rapid testing of tens to hundreds of conditions, and a 96‐well format enabling high‐throughput screening. These features make the IL‐2 WBA both before and after gluten challenge particularly suited for epitope mapping, pathogenesis studies and monitoring therapeutic effects on gluten‐specific T‐cell responses in pre‐clinical research. As the IL‐2 WBA is informative on the presence of and functional status of the gluten‐specific T cell in CD, it offers the ability to monitor these T cells without affecting or priming immune responses by stimulating with oral gluten challenge. More recently, we demonstrated that the IL‐2 WBA in a proprietary tube‐based format has promising diagnostic utility for CD, showing high sensitivity and specificity even in CD patients following a GFD, and particularly in HLA‐DQ2.5‐positive CD individuals.^24^ The tube‐based format may offer additional standardisation and ease of use suitable for clinical diagnostics or for use by clinical sites as part of CD therapeutic trial immunomonitoring.

This study has several limitations. First, the overall sample size was small. While the high sensitivity of the IL‐2 WBA is supported by other work,^24^, ^26^ the heterogeneity of immune responses to gluten in CD means that larger sample sizes would provide a better estimate of effect across the CD population. Second, the study focussed exclusively on HLA‐DQ2.5‐positive CD patients and assessed HLA‐DQ2.5‐restricted T‐cell epitopes. While this is the most common HLA genotype associated with CD (> 80%),^36^ the performance of the IL‐2 WBA assay using non‐DQ2.5 peptides needs further assessment.^12^ Third, while all CD patients had negative serology, dietary compliance was not objectively verified by quantification of gluten immunogenic peptides in stool or urine. Therefore, we cannot exclude the possibility that gluten exposure occurred prior to the study, which could impact IL‐2 responses. While we have shown here and in prior work that a single‐dose gluten challenge can ‘prime’ subsequent IL‐2 WBA responses by expanding the tetramer positive, gluten‐specific T‐cell pool,^24^ more sustained gluten exposure has been associated with reduced IL‐2 responses in the WBA.^24^, ^37^ Finally, several technical refinements could potentially enhance assay sensitivity. These include testing higher peptide concentrations beyond our current optimal dose, performing the ELISpot on fresh PBMC to allow a more direct comparison with the WBA, and implementing the S‐PLEX electrochemiluminescence kit. The S‐PLEX kit is approximately 12‐fold more sensitive, with a detection limit of 7.3 fg mL^−1^ compared to 90.0 fg mL^−1^ for the V‐PLEX kit used in this study.

In conclusion, the IL‐2 WBA demonstrates superior sensitivity compared to the IFN‐γ ELISpot in the detection and functional assessment of rare antigen‐specific T cells in circulation, both at baseline and after a single‐dose gluten challenge. It accurately reflects the systemic *in vivo* responses to gluten T‐cell epitopes and quantifies the effect of HLA blockade. The IL‐2 WBA enables T‐cell epitope mapping and immunomonitoring of antigen‐specific T‐cell responses with greater sensitivity than IFN‐γ ELISpot, and importantly, does not require patients to undergo gluten challenge. Beyond CD, the IL‐2 WBA shows promise as a versatile research and clinical tool across autoimmune, allergic, malignant, and infectious diseases where circulating antigen‐specific T cells are often present at low frequencies.

## Methods

### Study design and participants

This was a single centre, investigator‐led study performed at the Walter and Eliza Hall Institute, with recruitment via the Royal Melbourne Hospital. Adult participants aged 18–75 with medically diagnosed CD and NCGS were eligible to be recruited. All participants provided written informed consent. The study was approved by the Human Research Ethics Committees of the Royal Melbourne Hospital (2021.210) and the Walter and Eliza Hall Institute (21/18) and was conducted in accordance with the ethical principles in the Declaration of Helsinki.

Inclusion criteria for CD participants were the presence of documentation confirming a past CD diagnosis based on duodenal villous atrophy (Marsh 3), positive celiac serology and supportive clinical criteria. CD participants were following a GFD for at least 12 months and had negative transglutaminase‐IgA and deamidated gliadin peptide‐IgG (‘Treated CD’). Inclusion criteria for NCGS participants were self‐report of adverse symptoms to gluten, adherence to a GFD and documentation of prior exclusion of CD based on negative CD serology and/or small intestinal histology while eating gluten and/or the presence of an HLA genotype not consistent with CD. Exclusion criteria were the use of systemic immunosuppressant medication, pregnancy or the presence of refractory coeliac disease.

### Clinical procedures

Blood samples were collected from participants according to a standardised protocol: baseline (70 mL) on Day 1 prior to gluten challenge, followed by collections on Day 6 (120 mL) and Days 7 and 8 (100 mL each; CD participants only) after the administration of a single oral bolus of vital wheat gluten (10 g). Gluten challenge was undertaken as previously reported.^25^ Serum IL‐2 assessment required additional samples (5 mL) collected at baseline and 4 h post‐gluten challenge on Day 1 (‘GCIL‐2’). Clinical evaluation included coeliac‐specific serology (transglutaminase‐IgA and deamidated gliadin peptide‐IgG; Melbourne Health Pathology), medical history, current medications and patient‐reported outcome (PRO) measure using a modified CeD PRO^38^ questionnaire to document symptoms before, during and after gluten challenge.

### Peptides and antigens

Immunodominant gluten (gliadin) and hordein peptides including DQ2.5‐glia‐α1/α2, DQ2.5‐glia‐ω1/ω2, DQ2.5‐glia‐γ4e and DQ2.5‐Hor‐3a (Supplementary table 1) were synthesised to > 95% purity by high‐performance liquid chromatography, with liquid chromatography‐mass spectrometry identity confirmation (Mimotopes, Clayton, Australia). Peptides were solubilised in dimethyl sulfoxide (DMSO; Sigma‐Aldrich, MO, USA). As described elsewhere,^26^ peptide stock solutions were prepared in sterile phosphate buffered saline (PBS; Gibco, New York, NY, USA) as 10× (WBA) and 5× (ELISpot) final incubation concentrations and were dispensed in 25 μL aliquots to individual wells in sterile 96‐well U‐bottom microwell plates (Thermo Fisher Scientific, VIC, Australia). All conditions contained less than or equal to 0.05% DMSO, to ensure no toxicity.^39^ Plates were sealed with adhesive covers (MP Biomedicals, CA, USA) and stored at −80°C until use.

### Whole blood stimulation in microplates

Microplates containing peptides were thawed for 15–90 min at room temperature. Whole blood was collected via a 21 g needle into 10 mL Lithium–Heparin tubes (Becton Dickinson, NJ, USA), pooled and then dispensed (225 μL) into individual wells within 30 min of blood collection and incubated at 37°C/5% CO_2_ for 24 h ± 20 min. Three peptide concentrations were tested for α‐gliadin, ω‐gliadin, an equimolar pool of both α‐gliadin and ω‐gliadin (hereafter referred to as α + ω‐gliadin), and γ‐gliadin (0.2, 1 and 5 μg mL^−1^) and only the highest concentration was tested for Hor3a. PBS was used as a negative control. On Day 6 post gluten challenge, additional wells were tested with α‐gliadin or ω‐gliadin with 10 μg mL^−1^ SPVL3, an anti‐HLA‐DQ antibody, or with 10 μg mL^−1^ LB3‐1, an anti‐HLA‐DR antibody (kindly provided by Professor Anthony Purcell, Monash University). Each condition had four replicate wells, resulting in evaluation of approximately 0.5–2 million cells.^27^ Plasma was collected following separation by centrifugation at 1100 × *g* for 10 min, pooled and stored at −80°C until analysis.

### Electrochemiluminescence immunoassays

Electrochemiluminescence immunoassay kits (Meso Scale Discovery, MD, USA) were used for cytokine analysis according to the manufacturer's instructions. IL‐2 was measured in 0 and 4 h serum using S‐PLEX kits and IL‐2, IFN‐γ and IL‐10 in plasma from WBA were measured using customised V‐PLEX Proinflammatory panel kits. Plates were run on a SQ120MM instrument. Mean cytokine levels from duplicate wells were analysed using MSD Discovery Workbench software. Fold change was calculated by dividing the IL‐2 concentration from 4‐h serum by 0‐h serum for GCIL‐2 or peptide‐stimulated plasma by PBS‐stimulated plasma from WBA, and a twofold change cut‐off was applied as previously established.^12^ Total IL‐2 concentration was reported after subtracting 0‐h serum from 4‐h serum or PBS plasma from peptide plasma. The lower limit of quantification (LLOQ) was calculated in the software for each assay plate. Cytokine concentrations were only below the LLOQ for IL‐2; thus, the LLOQ is indicated only in figures showing IL‐2 concentration, and values below the LLOQ were substituted with the LLOQ when calculating fold change.

### IFN‐γ ELISpot

IFN‐γ ELISpot assays were performed and analysed as previously described.^16^, ^17^, ^30^ Briefly, peripheral blood mononuclear cells (PBMC) were isolated from heparinised whole blood by Ficoll‐Paque™ Plus (GE Healthcare, NJ, USA) density‐gradient centrifugation (Leucosep tubes; Interpath Services, VIC, Australia) and cryopreserved. PBMC were thawed and resuspended to a concentration of 2.5–5 million PBMC mL^−1^ in complete RPMI‐1640 supplemented with heat‐inactivated 10% pooled human serum (Lifeblood, VIC, Australia), 1× GlutaMAX (Gibco), 1× non‐essential amino acids (Gibco) and 50 μm 2‐mercaptoethanol (Sigma‐Aldrich) and were passed through a 70 μm cell strainer. Pre‐coated IFN‐γ ELISpot Plus plates (Mabtech, Nacka Strand, Sweden) were blocked with 200 μL of RPMI + 10% fetal bovine serum (HyClone Cytiva, UT, USA) for 1–2 h at 37°C. Peptides were thawed and 25 μL dispensed into duplicate wells of the ELISpot plate with 100 μL of PBMC (0.25–0.5 million per well) and incubated overnight at 37°C in 5% CO_2_. These conditions result in the evaluation of approximately 0.5–1 million cells, similar to the whole blood stimulation. The detection antibodies and solution were added as per the manufacturer's instructions. Spot forming units (SFU) in individual wells were counted using an automated ELISpot reader (AID ELISpot Reader System; AID Autoimmun Diagnostika GmbH, Strassberg, Germany) and results expressed as mean SFU per million PBMC. A response cutoff was determined as the mean SFU for the PBS control from all patients at all time points +3 standard deviations.

### Statistics

Data were analysed using GraphPad Prism (version 5). Nonparametric statistical tests were used to compare paired data (Friedman or Wilcoxon) and unpaired data (Mann–Whitney) with correction for multiple comparisons where applicable.

## Author contributions

**Olivia G Moscatelli:** Conceptualization; writing – original draft; writing – review and editing; methodology; formal analysis. **Amy K Russell:** Investigation; writing – review and editing. **Lee M Henneken:** Resources; writing – review and editing. **Melinda Y Hardy:** Supervision; writing – review and editing; investigation. **Jason A Tye‐Din:** Writing – original draft; writing – review and editing; supervision; funding acquisition; conceptualization.

## Conflict of interest

MYH is a consultant for Takeda. JAT‐D has privately or via his institute been a consultant or advisory board member for Anatara, Anokion, Barinthus Biotherapeutics, Chugai Pharmaceuticals, DBV Technologies, Dr Falk, EVOQ Therapeutics, Equillium, Forte Biosciences, IM Therapeutics, Janssen, Kallyope, Mozart Therapeutics, Takeda, TEVA and Topas, has received research funding from Barinthus Biotherapeutics, Chugai Pharmaceuticals, Codexis, DBV Technologies, Kallyope, Novoviah Pharmaceuticals, Sonoma Biotherapeutics, Topas and Tillotts Pharmaceuticals. He is an inventor on patents relating to the use of gluten peptides in celiac disease diagnosis and treatment. All other authors have no conflicts to declare.

## Supporting information


Supplementary figures 1–3
Supplementary table 1

## Data Availability

The data that support the findings of this study are available from the corresponding author upon reasonable request.
