# Supplementary material for: An optimised whole blood interleukin‐2 release assay is more sensitive than interferon‐γ ELISpot for detecting and quantifying gluten‐specific CD4+ T‐cell responses in coeliac disease
Source: Clin Transl Immunology. 2025 Dec 10;14(12):e70063. doi: 10.1002/cti2.70063 (PMC12695692; doi:10.1002/cti2.70063)
Supplement: Supplementary file 1 — Supplementary figures 1–3 Supplementary table 1 [file CTI2-14-e70063-s001.docx]

**Supplementary Materials**

**Supplementary Table 1: Peptide sequences**

| Peptide list | Epitope name | Amino acid sequence |
| --- | --- | --- |
| α-gliadin | DQ2.5-glia-α1/α2 | ZLQPFPQPELPYPQPQ-NH_2_ |
| ω-gliadin | DQ2.5-glia-ω1/ω2 | ZQPFPQPEQPFPWQP-NH_2_ |
| γ-gliadin | DQ2.5-glia-γ4e | ZPFPLQPEQPFPQP-NH_2_ |
| Hor3a | DQ2.5-Hor-3a | ZPEQPIPEQPQPYPQQ-NH_2_ |

**
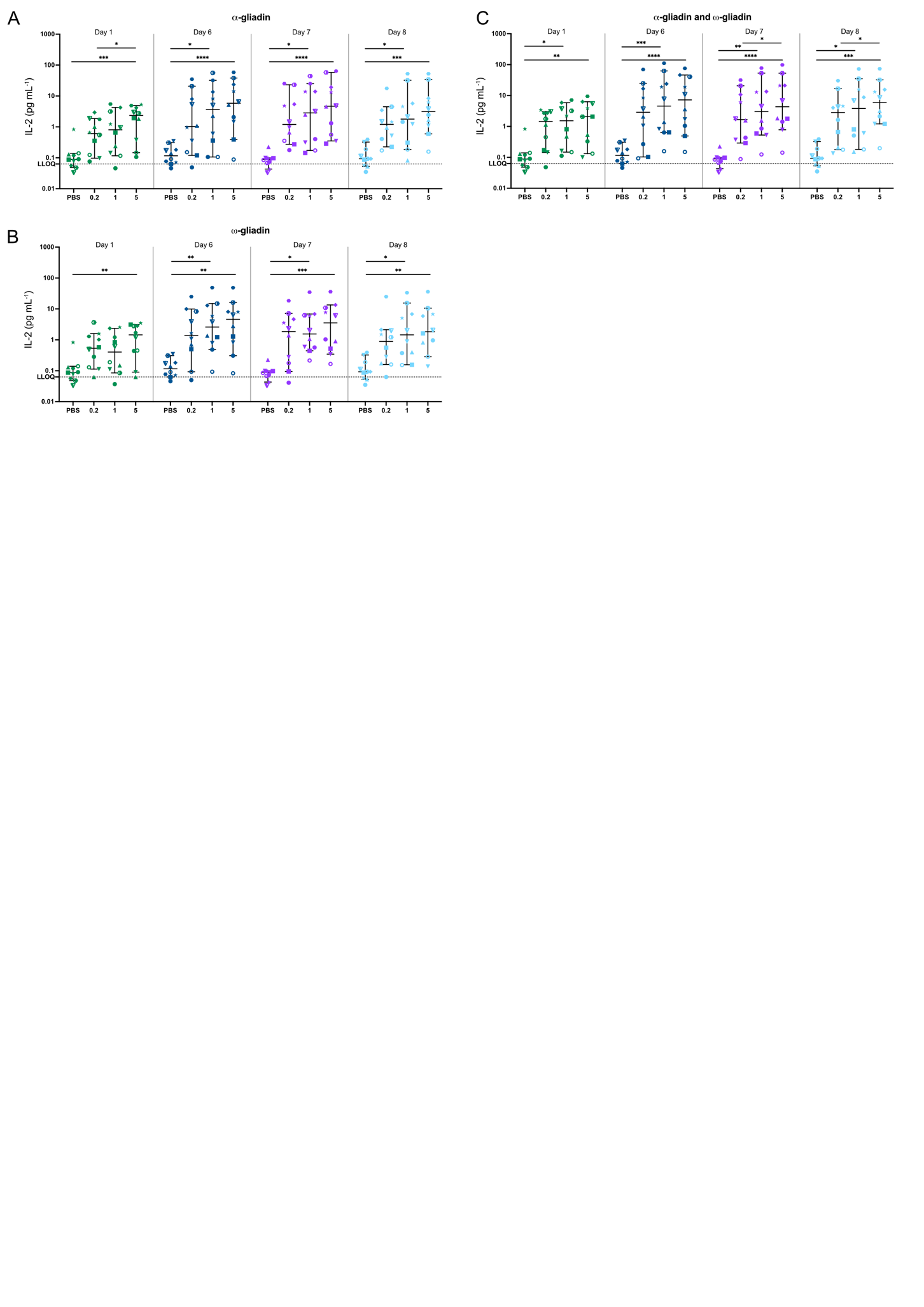
Supplementary Figure 1: IL-2 WBA responses across concentrations and days in treated CD**

IL-2 concentration (pg mL^-1^) are shown from WBA stimulation with PBS or 0.2, 1 or 5 μg mL^-1^ of α-gliadin (**A**), ω-gliadin (**B**), and an equimolar pool of α-gliadin and ω-gliadin (**C**) in treated coeliac disease (CD) participants (n = 10) at baseline (day 1) and days 6-8 post gluten challenge. Median and 95% confidence intervals are shown. Dashed lines indicate lower limit of quantification (LLOQ). Friedman test was performed to compare peptide concentrations; **P* < 0.05, ***P* < 0.01, ****P* < 0.001.

**
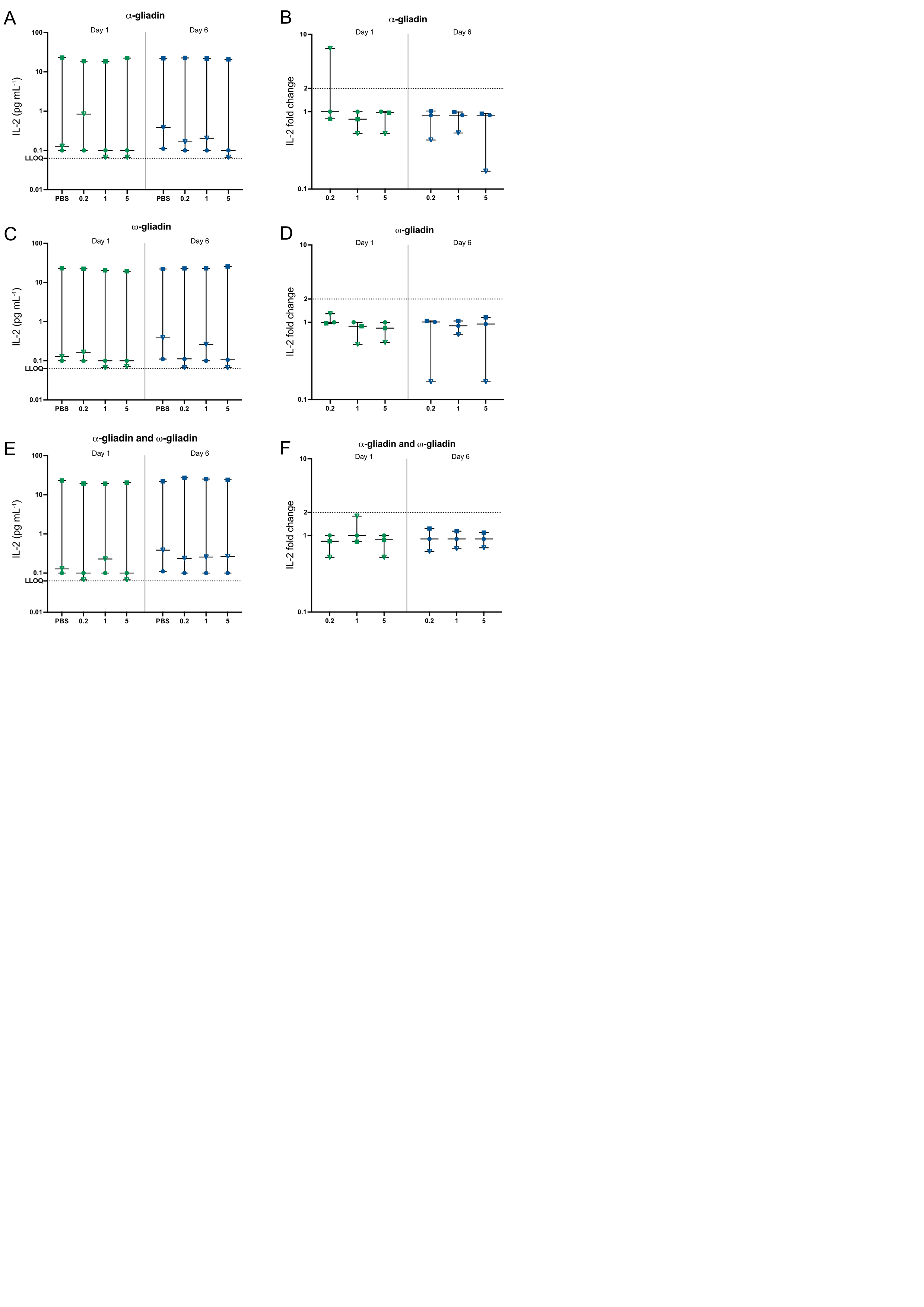
Supplementary Figure 2: IL-2 WBA responses across concentrations and days in NCGS**

IL-2 concentration (pg mL^-1^; left) and fold change (right) are shown from WBA stimulation with PBS or 0.2, 1 or 5 μg mL^-1^ of α-gliadin (**A, B**), ω-gliadin (**C, D**) and an equimolar pool of α-gliadin and ω-gliadin (**E, F**) in non-coeliac gluten sensitive (NCGS) participants (n = 3) at baseline (day 1) and day 6 post gluten challenge. Dashed lines indicates lower limit of quantification (LLOQ; left) or 2-fold change cutoff (right). Median and 95% confidence intervals are shown. Friedman test was performed to compare peptide concentrations.

**
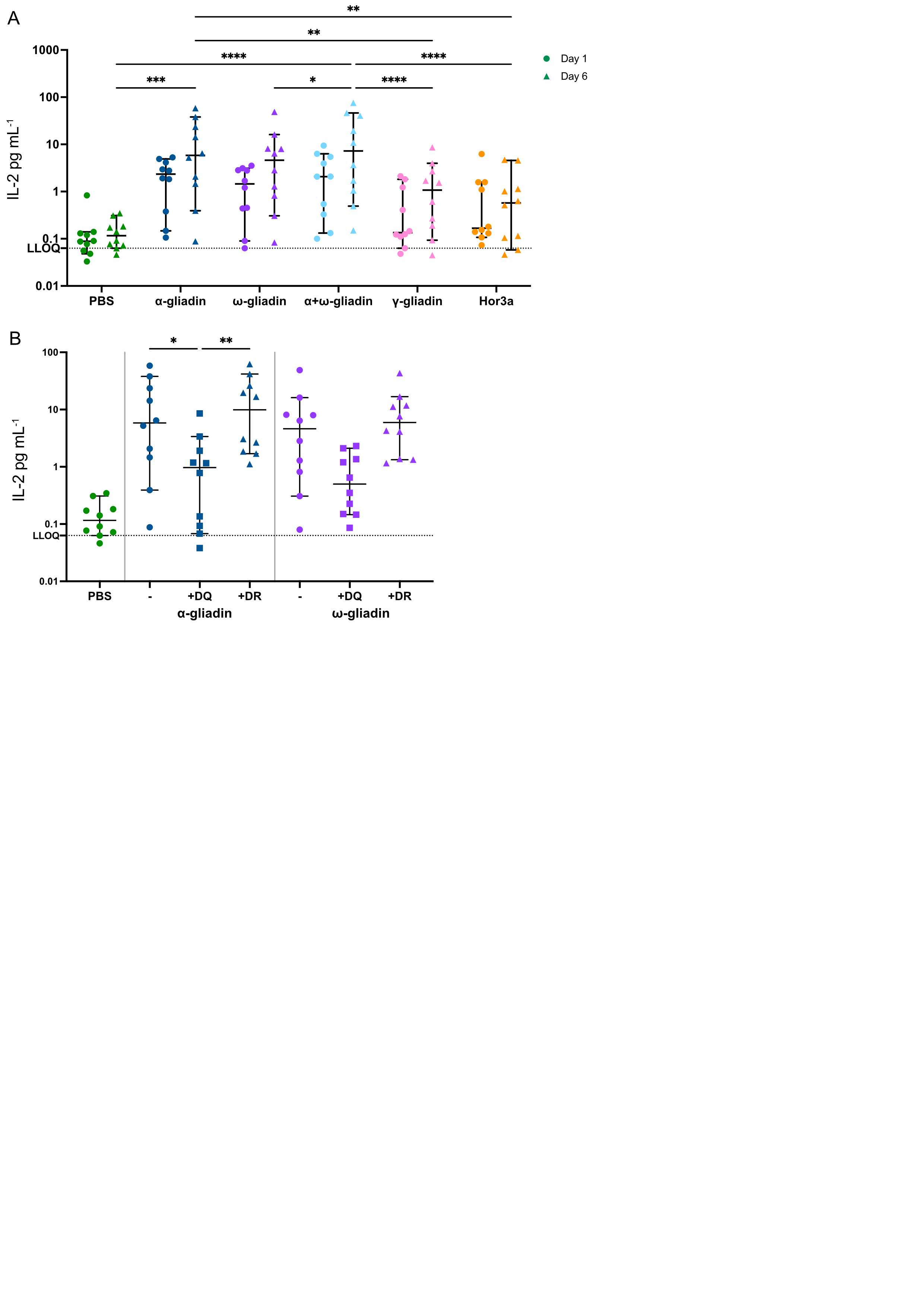
Supplementary Figure 3: IL-2 WBA reflects epitope hierarchy and immune modulation**

IL-2 concentration (pg mL^-1^) from WBA stimulation with 5 μg mL^-1^ of peptide in treated coeliac disease (CD) participants (n = 10) at baseline (circle) and day 6 (triangle) post gluten challenge (**A**). Responses to stimulation with α-gliadin, ω-gliadin, α-gliadin and ω-gliadin, γ-gliadin, and hor3a are shown. Responses to stimulation with PBS (green), α-gliadin (blue) or ω-gliadin (purple) alone (-, circle), with anti-HLA-DQ (+DQ, square), or with anti-HLA-DR (+DR, triangle) are shown (**B**). Dashed lines indicate lower limit of quantification (LLOQ). Median and 95% confidence intervals are shown. Friedman test was performed to compare response to different stimulation conditions; ***P* < 0.05, ***P* < 0.01, ****P* < 0.001.
